# A Pax3/Dmrt2/Myf5 Regulatory Cascade Functions at the Onset of Myogenesis

**DOI:** 10.1371/journal.pgen.1000897

**Published:** 2010-04-01

**Authors:** Takahiko Sato, Didier Rocancourt, Luís Marques, Sólveig Thorsteinsdóttir, Margaret Buckingham

**Affiliations:** 1 Department of Developmental Biology, Centre National de la Recherche Scientifique, Unité de Recherche Associée 2578, Institut Pasteur, Paris, France; 2 Department of Animal Biology and Centre for Environmental Biology, Faculty of Sciences, University of Lisbon, Lisbon, Portugal; 3 Gulbenkian Institute of Science, Oeiras, Portugal; Medical Research Council Human Genetics Unit, United Kingdom

## Abstract

All skeletal muscle progenitor cells in the body derive from the dermomyotome, the dorsal epithelial domain of developing somites. These multipotent stem cells express Pax3, and this expression is maintained in the myogenic lineage where Pax3 plays an important role. Identification of Pax3 targets is therefore important for understanding the mechanisms that underlie the onset of myogenesis. In a microarray screen of Pax3-GFP sorted cells, with analysis on *Pax3* gain and loss of function genetic backgrounds, we identify *Dmrt2*, expressed in the dermomyotome, as a Pax3 target. *In vitro* gel shift analysis and chromatin immunoprecipitation with *in vivo* extracts show that Pax3 binds to a conserved 286 bp sequence, situated at −18 kb from *Dmrt2*. This sequence directs reporter transgene expression to the somite, and this is severely affected when the Pax3 site is mutated in the context of the locus. In *Dmrt2* mutant embryos, somite maturation is perturbed and the skeletal muscle of the myotome is abnormal. We now report that the onset of myogenesis is also affected. This depends on activation, in the epaxial dermomyotome, of the myogenic determination gene, *Myf5*, through its early epaxial enhancer. This sequence contains sites that bind Dmrt2, which belongs to the DM class of DNA–binding proteins. Mutation of these sites compromises activity of the enhancer in transgenic embryos where the reporter transgene is under the control of the *Myf5* epaxial enhancer. Transactivation of this site by Dmrt2 is demonstrated *in vitro*, and conditional overexpression of Dmrt2 in *Pax3* expressing cells in the somite confirms the role of this factor in the activation of *Myf5*. These results reveal a novel genetic network, comprising a *Pax3/Dmrt2/Myf5* regulatory cascade that operates in stem cells of the epaxial dermomyotome to initiate skeletal muscle formation.

## Author Summary

It is well established that skeletal muscle derives from segmented structures called somites that form on either side of the axis of the embryo. The part of the somite that contains muscle stem cells is called the dermomyotome. These cells express the transcription factor Pax3, which regulates muscle stem cell behaviour. We now show that the *Dmrt2* gene, also expressed in the dermomyotome, is directly controlled by Pax3. Since Dmrt2 has been implicated in maintaining the integrity of the dermomyotome, this therefore indicates an upstream role for Pax3 in this structure as well as in controlling cells that form skeletal muscle. Furthermore Dmrt2 directly regulates early activation of the myogenic determination gene, *Myf5*, required for the formation of the first skeletal muscle in the somite. This is a novel function for Dmrt2 and shows that this transcription factor controls both structure and cell fate. Our results reveal a *Pax3/Dmrt2/Myf5* regulatory cascade through which Pax3 orchestrates the onset of myogenesis in the muscle stem cells of the dermomyotome.

## Introduction

The Pax family of transcriptional regulators play key roles in the onset of organogenesis and cell lineage specification during development [1]. Pax3 and Pax7 regulate skeletal muscle formation. Skeletal muscle progenitors in the trunk and limbs of vertebrates derive from somites, from the dorsal compartment known as the dermomyotome. In the mouse embryo, Pax3 is expressed throughout this epithelial structure, whereas Pax7 expression is restricted to the central domain. Myogenesis is initiated by the delamination of Pax3 positive cells from the edges of the dermomyotome; at certain axial levels, cells from the hypaxial domain migrate from the somite, before activating the myogenic determination genes *Myf5* and *MyoD*, whereas other cells, most notably in the epaxial domain, have already activated *Myf5* and immediately differentiate, on delamination, to form the first skeletal muscle of the epaxial myotome. Transcription of *Myf5* at this site depends on an early epaxial enhancer, which lies at −5.5 kb from the gene [2],[3]. This sequence is regulated by Shh [4]–[6] and by canonical Wnt signalling [6] from the adjacent axial structures, acting through Gli and TCF binding sites. Subsequently, as the somite matures, the central dermomyotome loses its epithelial structure and cells that are positive for Pax3 and Pax7 enter the underlying muscle masses. These provide an essential population of muscle stem cells for all subsequent muscle growth. In *Pax3^−/−^*; *Pax7^−/−^* double mutants, these cells fail to activate myogenic determination genes and many of them die [7]. In the absence of Pax3 alone, cell death is also observed at the extremities of the dermomyotome, where it is most evident in the hypaxial domain [1].

Pax3 target genes that are important for the onset of myogenesis are beginning to be identified. A classic example is provided by the gene encoding c-Met, a receptor required for delamination and migration of muscle progenitor cells [8]. More recently, a *Myf5* regulatory sequence required for activation of this myogenic determination gene in the limb and hypaxial somite has been identified as a direct Pax3 target [9]. The second myogenic determination gene, *MyoD*, has been shown to be a target of Pax3/7 in a myogenic cell line and in postnatal muscle progenitor cells [10] and indeed other Pax7 [11],[12] targets have been identified in this cellular context. Activation of myogenic determination genes leads to entry into the myogenic programme, which is accompanied by down-regulation of Pax3/7, to which microRNAs contribute [13]. However, maintenance of a muscle stem cell population is essential in the growing organism and this is achieved through signalling systems that maintain a balance between self-renewal and differentiation [14]. In the context of FGF signalling, Pax3 directly regulates *Fgfr4* and acts genetically upstream of other components of the pathway also, such as *Sprouty1*, to control self-renewal versus differentiation of Pax3 positive muscle progenitors [15].

In order to identify further Pax3 target genes acting at earlier stages within the dermomyotome, we have performed a microarray screen that has led to the identification of *Dmrt2*. This gene family was first characterized as *doublesex* in *Drosophila* [16]–[18] and as *mab-3* in *C.elegans* [19], where these genes play important roles in sex determination [20],[21]. Vertebrate homologues have since been identified, some of which also play a role in sexual development [22]. However *Dmrt* genes are also expressed outside the gonads and have been implicated in other developmental processes. *Dmrt2*/*Terra* is expressed during somitogenesis in vertebrates [23], where transcripts are present in the presomitic mesoderm (PSM) and then confined to the dermomyotome of somites. In the chick embryo, *Terra* is expressed symmetrically in the PSM, but has a transient asymmetrical expression around the node, implicating it in left-right axis determination and normal development of bilateral somites through interaction with the segmentation clock, as indicated by morpholino experiments in zebrafish [24]. Mouse embryos lacking Dmrt2 show somite patterning defects, culminating in malformed ribs and sternum leading to postnatal death due to respiratory problems [25]. Such malformations are often associated with abnormal dermomyotome and myotome development [1] and indeed *Dmrt2^−/−^* embryos have morphological defects in these somite compartments, with perturbation of the expression of myogenic markers reported at E10.5 and E11.5. In *Dmrt2* and *Pax3* compound mutants perturbations in myogenesis were also observed [26], as evidenced by muscle differentiation markers at E10.5. However cell death in the absence of Pax3 [1] complicates the interpretation.

The molecular function of Dmrt2 has not been investigated. Dmrt proteins are characterised by the DM domain, an intertwined Zn finger-like motif that interacts with the minor groove of DNA [27]. The conserved DM domains of mammalian Dmrt factors bind to similar DNA consensus sequences [28]. *Drosophila* Dsx^m^ acts as a transcriptional activator whereas Dsx^F^ has repressor function [29]. When human DMRT1 is fused to the VP16 activation domain, transactivation through the consensus DMRT1 binding sequence is observed, however DMRT1 alone did not show much activity [28]. Regulation of the *Dmrt2* gene, present in a cluster with *Dmrt1, 3* in mice, has not been examined, although the human *DMRT2* gene has been shown to encode alternatively spliced transcripts [30].

In this report, we identify *Dmrt2* as a target of Pax3, acting directly through a regulatory sequence that directs expression in the somite. We show that Dmrt2, in turn, acts on the epaxial enhancer of *Myf5*. Perturbation of this regulatory network, functioning in the dermomyotome, has consequences for the onset of myogenesis.

## Results

In a screen designed to detect Pax3 targets in the dermomyotome of interlimb somites at E9.5, this region of the somite was dissected from *Pax3^GFP/+^* heterozygote and *Pax3^PAX3-FKHR-IRESnlacZ/GFP^* gain of function embryos [31]. After dissociation, GFP positive cells were separated by flow cytometry. Microarray comparisons of the two classes of samples led to identification of a series of sequences that are up- or down-regulated in the presence of PAX3-FKHR, a constitutively active form of the Pax3 transcription factor [31]. Among these sequences, *Dmrt2* was up-regulated 1.87 fold (data not shown).

**Figure 1:**
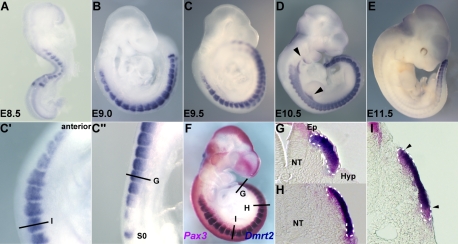
The expression of *Dmrt2*, visualised by whole mount *in situ* hybridization. Embryos were hybridized with a *Dmrt2* RNA probe. (A) an E8.5 embryo, (B) an E9.0 embryo, (C, F) E9.5 embryos, (D) an E10.5 embryo, (E) an E11.5 embryo. C′ and C″ are magnified views of C. *Dmrt2* transcripts are present in all somites until E11.5 when they are only detected caudally. (F) A *Pax3^IRES-nlacZ/+^* embryo at E9.5 hybridized with a *Dmrt2* probe (blue) and treated with Red-gal to reveal β-galactosidase (β-gal) expression from the *Pax3^IRES-nlacZ^* allele (red). *Dmrt2* expression was also detected in proximal forelimb buds and branchial arches (arrowheads in D). (G–I) Transverse sections of the embryos shown in F. Equivalent axial levels are also indicated in C′ and C″. White dotted lines outline the dermomyotome. *Dmrt2* expression is observed in the epaxial domain of immature somites (C″, G), and then throughout the Pax3 positive dermomyotome (C′, H). In more mature anterior somites, the expression of *Dmrt2* in the epaxial and hypaxial edges of the dermomyotome has decreased (I). S0, presomitic mesoderm prior to the first somite; NT, neural tube; Ep, epaxial somite; Hyp, hypaxial somite.

*Dmrt2* is transcribed in mouse somites (Figure 1), as previously reported [25]. Expression is detected as a band in presomitic mesoderm (S0) and in the first somites at E8.5, where transcripts accumulate in the epaxial domain, as shown for immature caudal somites at E9.5 (C″). Sections of a *Pax3^IRESnlacZ/+^* embryo at this stage (Figure 1F) show that *Dmrt2* expression is confined to the Pax3 positive dermomyotome of the somite, in the epaxial domain of the most immature caudal somites (Figure 1G), and then throughout this epithelium in more mature somites (Figure 1H). Already, at this stage, the most mature anterior somites (Figure 1I) are beginning to lose *Dmrt2* expression at the epaxial and hypaxial extremities of the dermomyotome. This is detected also by whole mount *in situ* hybridization at E9.5 (Figure 1C and 1C′) and at E10.5 (Figure 1D), when labelled cells are also detectable in the mesodermal core of the 1st and 2nd branchial arches and in the forelimb bud, to which myogenic progenitor cells have begun to migrate from the somites [1]. By E11.5 (Figure 1E), *Dmrt2* transcripts are only detectable in caudal somites.

**Figure 2:**
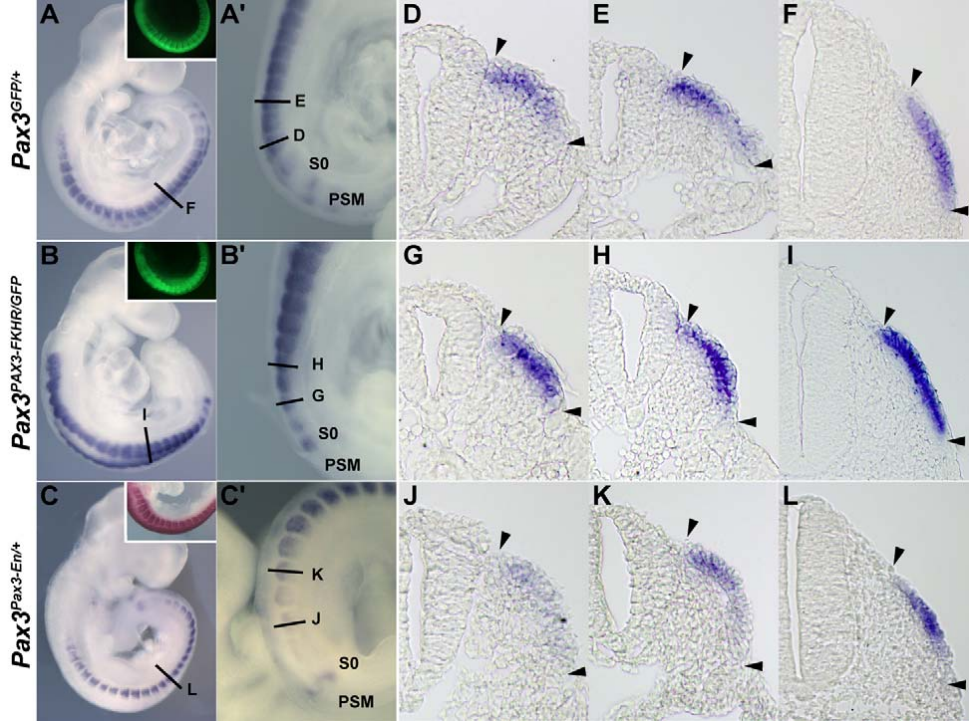
The expression of *Dmrt2* is controlled by *Pax3*. (A–C) Whole mount *in situ* hybridization with a *Dmrt2* probe of *Pax3^GFP/+^* (A), *Pax3^PAX3-FKHR-IRESnlacZ/GFP^* (*Pax3^PAX3-FKHR/GFP^*, B) and *Pax3^Pax3-En-IRESnlacZ/+^* (*Pax3^Pax3-En/+^*, C) embryos at E9.5. *Pax3* expression is shown as GFP fluorescence (inserts in A, B), or as Salmon-gal staining (insert in C) of trunk somites. A′–C′ show enlargements of the posterior somite regions used for sections. (D–L) Sections at equivalent axial levels showing *Dmrt2* transcripts in the dermomyotome of control *Pax3^GFP/+^* (D–F), gain of function *Pax3^PAX3-FKHR/GFP^* (G–I) or partial loss of function *Pax3^Pax3-En/+^* (J–L) embryos. *Dmrt2* transcripts are higher in gain of function embryos, where they are now detectable throughout the dermomyotome, whereas when Pax3 activity is altered they are barely detectable in the epaxial domain of immature somites (J) and detectable in a restricted region of more mature somites (L). Arrowheads indicate the full extent of the dermomyotome. PSM, presomitic mesoderm; S0, PSM prior to the first somite.

The expression of *Dmrt2* is similar in *Pax3^GFP/+^* heterozygote embryos (Figure 2A and 2D–2F) to that in wild type embryos (Figure 1). However *Dmrt2* expression levels are higher in the dermomyotome of *Pax3^PAX3-FKHR-IRESnlacZ/GFP^* gain of function embryos (Figure 2B and 2G–2I), whereas they are notably lower in *Pax3^Pax3-En-IRESnlacZ/+^* embryos, where the presence of a fusion protein in which the DNA binding domain of Pax3 is fused to the repression domain of Engrailed, results in a partial loss of function phenotype [9], attenuating the cell death seen in the absence of Pax3. Diminution of *Dmrt2* expression is also seen in *Pax3* mutant embryos at E9.25 (Figure S1), prior to extensive cell death. Maintenance of *Dmrt2* expression in the central dermomyotome may reflect the expression of *Pax7* in this domain. These results confirm that *Dmrt2* lies genetically downstream of *Pax3*.

**Figure 3:**
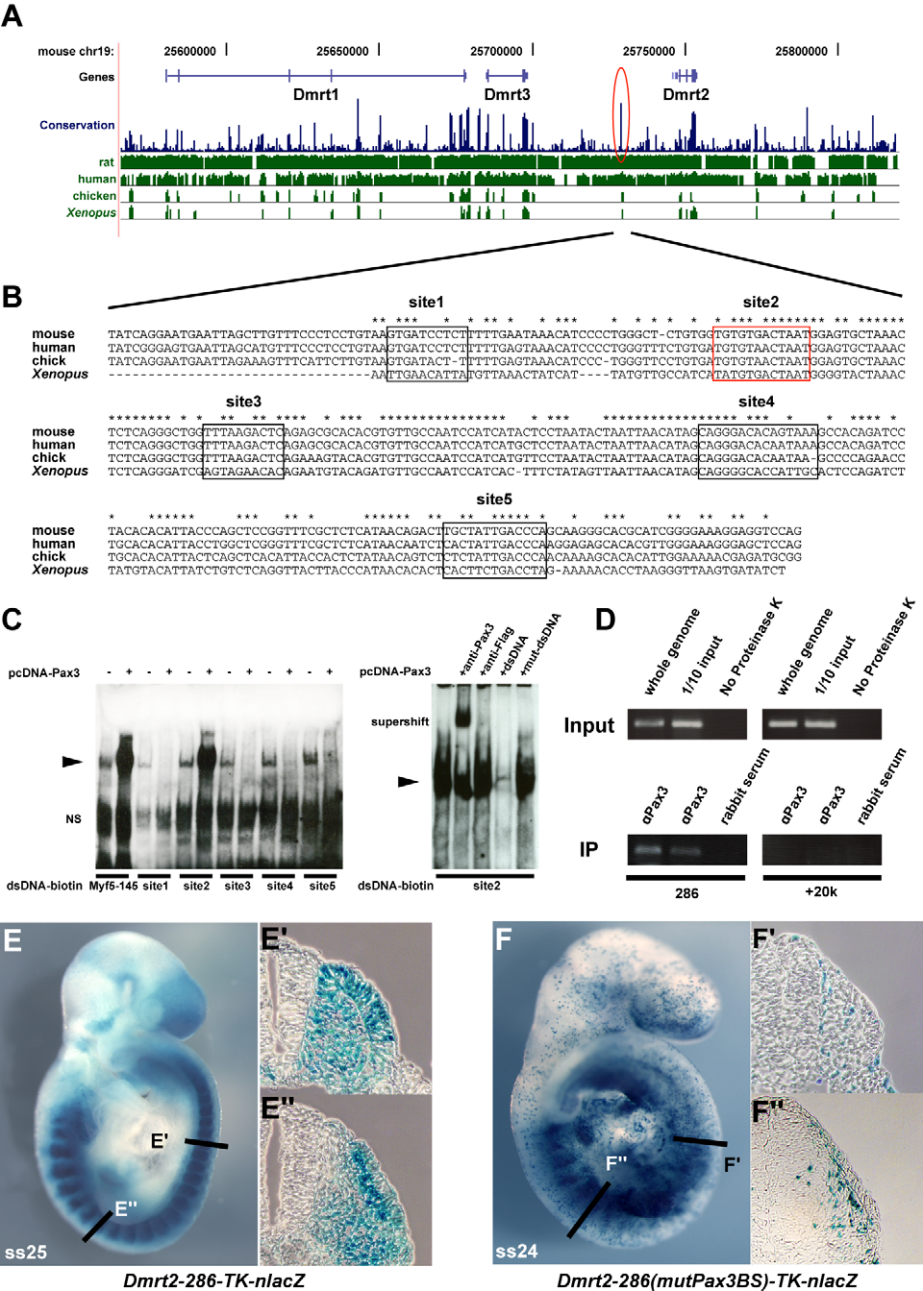
A conserved 286 bp element 5′ of the *Dmrt2* gene directs Pax3-dependent expression in the somite. (A) A schematic representation of the mouse *Dmrt* genomic locus showing overall sequence conservation (blue bars) between human, rat, chicken and *Xenopus* (green bars). (B) The nucleotide sequence of the conserved 286 bp *Dmrt2* element (red circle in A) in mouse (the position on ch19; 25728951 to 25729236, about 18 kb 5′ of *Dmrt2*) and comparison with a homologous region of human, chick and *Xenopus* genomes, with asterisks indicating conserved nucleotides between all these species. Five putative Pax3 binding sites are framed in black and red. (C) Electrophoretic mobility shift assays (EMSA) performed with oligonucleotides conjugated with biotin, containing putative Pax3 binding sites (1–5) as indicated in (B), incubated with extracts of HEK293 cells, with (+) or without (−) a Pax3 expression vector, or oligonucleotides without biotin (+dsDNA), including mutated oligonucleotides (+mut-dsDNA). The Pax3 site in the 145 bp regulatory element at −57.5 kb from the *Myf5* gene provides a positive control (Myf5-145; [9]). Significant binding is seen on site2 when Pax3 protein is present. For supershift assays, monoclonal Pax3 antibody (DSHB) was used (+anti-Pax3); +anti-Flag provides a negative control. Arrowheads show the band due to binding of the Pax3 protein. NS; non-specific. (D) Chromatin immunoprecipitation (ChIP) analysis of Pax3 binding to the 286 bp sequence *in vivo* was performed with chromatin prepared from somites of E9.5 embryos (without head, neural tube, and internal tissues). Upper panels show the evaluation of sheared genomic DNA (1/10 input) with two sets of *Dmrt2* primers, for the potential regulatory sequence (286) and for an upstream control sequence (+20 k) without Pax3 binding sites (results not shown), in left and right panels respectively. As an additional positive or negative control, whole genome sheared genomic DNA, with (whole genome) or without proteinase K (No Proteinase K) treatment is shown (In the absence of proteinase K cross-linked chromatin blocks the PCR reaction). Lower panels show ChIP (IP) with two different Pax3 antibodies (αPax3, Bajard et al. (2006), Lagha et al. (2008)) and control rabbit serum. Left lower bands indicate Pax3 binding to the 286 bp sequence, not seen with the control +20 kb sequence (right panels). (E) A transient transgenic embryo (E9.5) with the 286 bp *Dmrt2* fragment, fused to the *TK* promoter and *nlacZ* reporter shows dermomyotome expression. (F) The transgene, with mutated Pax3 binding site2, shows loss of β-galactosidase activity in the dermomyotome (F′), but some ectopic expression in the myotome and ventral somite (F″), seen in more mature somites in the non-mutated transgenic embryo (E″). Observations on transgenic embryos are summarised in Table 1.

In order to see whether *Dmrt2* is a direct Pax3 target, we examined the *Dmrt2* locus on mouse chromosome 19, which includes other *Dmrt* genes (1, 3), for non-coding sequences that are conserved between species (Figure 3A). A conserved 286 bp region at about −18 kb from the *Dmrt2* gene attracted our attention. This region has five putative Pax3 binding sites (Figure 3B). Electrophoretic mobility gel shift assays with oligonucleotides encompassing these sites and *in vitro* synthesized Pax3 protein showed that site2 binds Pax3, a result confirmed by a super-shift experiment with a Pax3 antibody (Figure 3C). Chromatin immunoprecipitation of embryo extracts at E9.5 demonstrated that Pax3 binds specifically to the 286 bp sequence at −18 kb from the *Dmrt2* gene *in vivo*. The function of the 286 bp sequence was tested in transgenic embryos. It directs transgene expression in the dermomyotome, where the endogenous gene is expressed, with additional ectopic expression in the ventral somite (Figure 3E). Mutation of Pax3 site2 in the 286 bp sequence interferes with the expression of the transgene in the dermomyotome (Figure 3F). Other conserved regions, notably at +20 kb and +37 kb did not direct reporter gene expression in transgenic embryos. A BAC transgenic analysis had shown that 150 kb of 5′ and 50 kb of 3′ genomic sequence flanking the *Dmrt2* gene directed somitic expression in transgenic embryos. When the Pax3 binding site in the 286 bp sequence was mutated in the context of this BAC, somitic expression was severely affected (Figure S2). This result therefore indicates that the Pax3 site within this sequence plays an important role in the regulation of the *Dmrt2* gene.

**Figure 4:**
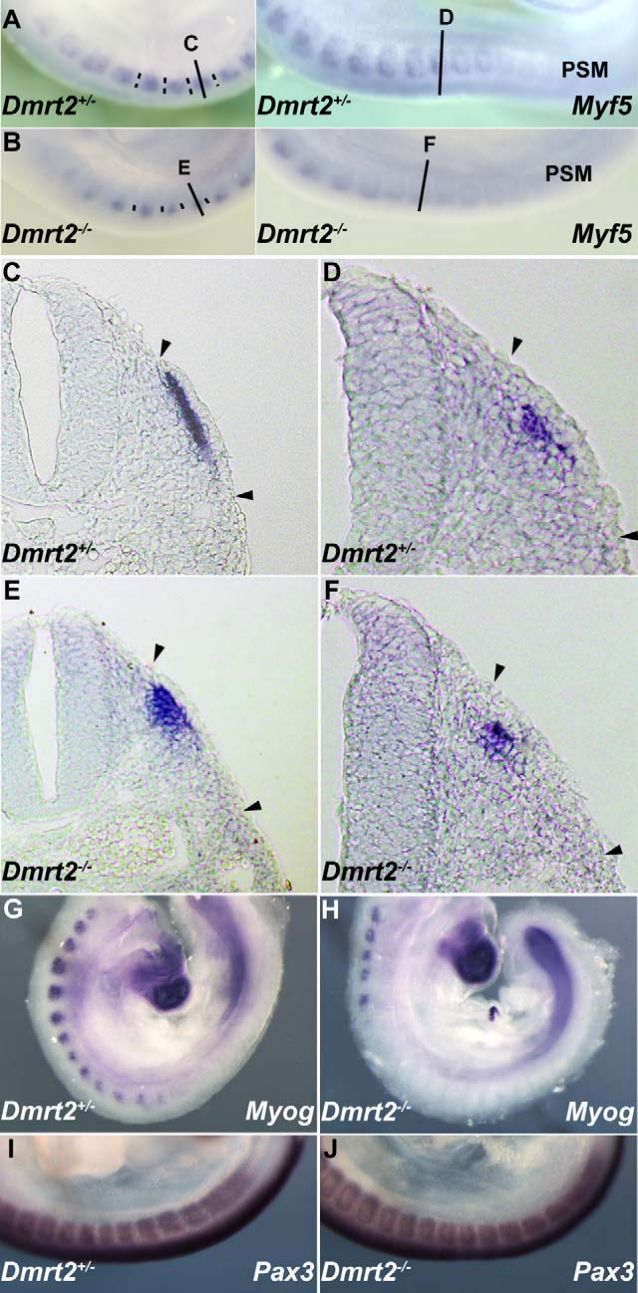
Dmrt2 is required for the maturation of the epaxial somite, where *Myf5* is first expressed. Whole mount *in situ* hybridization of *Dmrt2^+/−^* (A) and *Dmrt2^−/−^* (B) embryos at E9.5, with a *Myf5* probe. (C–F) Sections of embryos shown in (A, B) at different axial levels - left hand panels show more mature somites; immature somites where the epaxial myotome is beginning to form are shown in right hand panels. The onset of *Myf5* expression is perturbed in the absence of Dmrt2. (G, H) Whole mount *in situ* hybridization of *Dmrt2^+/−^* (G) and *Dmrt2^−/−^* (H) embryos with a *myogenin* (Myog) probe, showing a striking delay of *myogenin* expression in the absence of Dmrt2. (I, J) Whole mount *in situ* hybridization of *Dmrt2^+/−^* (1) and *Dmrt2^−/−^* (J) embryos showing *Pax3* transcripts.

Dmrt2 has been implicated in somite patterning, with morphological abnormalities reported in mature somites from E10.5 [25]. These include abnormal expression of myotomal markers. Since *Dmrt2* is first expressed in the epaxial dermomyotome where activation of the myogenic determination gene, *Myf5*, initiates the onset of myogenesis [1], we examined *Myf5* expression in *Dmrt2* mutant embryos. *Myf5* activation in the epaxial domain is retarded in the absence of Dmrt2. This is detectable on whole mount *in situ* hybridization (Figure 4A and 4B). On sections of somites at equivalent axial levels, *Myf5* expression is reduced. The delay in the colonisation of the myotome reflects the reduction in *Myf5* transcription in the epaxial domain. (Figure 4D and 4F). This perturbation in *Myf5* activation has consequences for myogenesis, as evidenced by the delay in expression of the gene for the myogenic differentiation factor, myogenin (Figure 4G and 4H), in the absence of Dmrt2. *Pax3* transcription, on the other hand, is not reduced at the onset of somitogenesis (Figure 4I and 4J).

**Figure 5:**
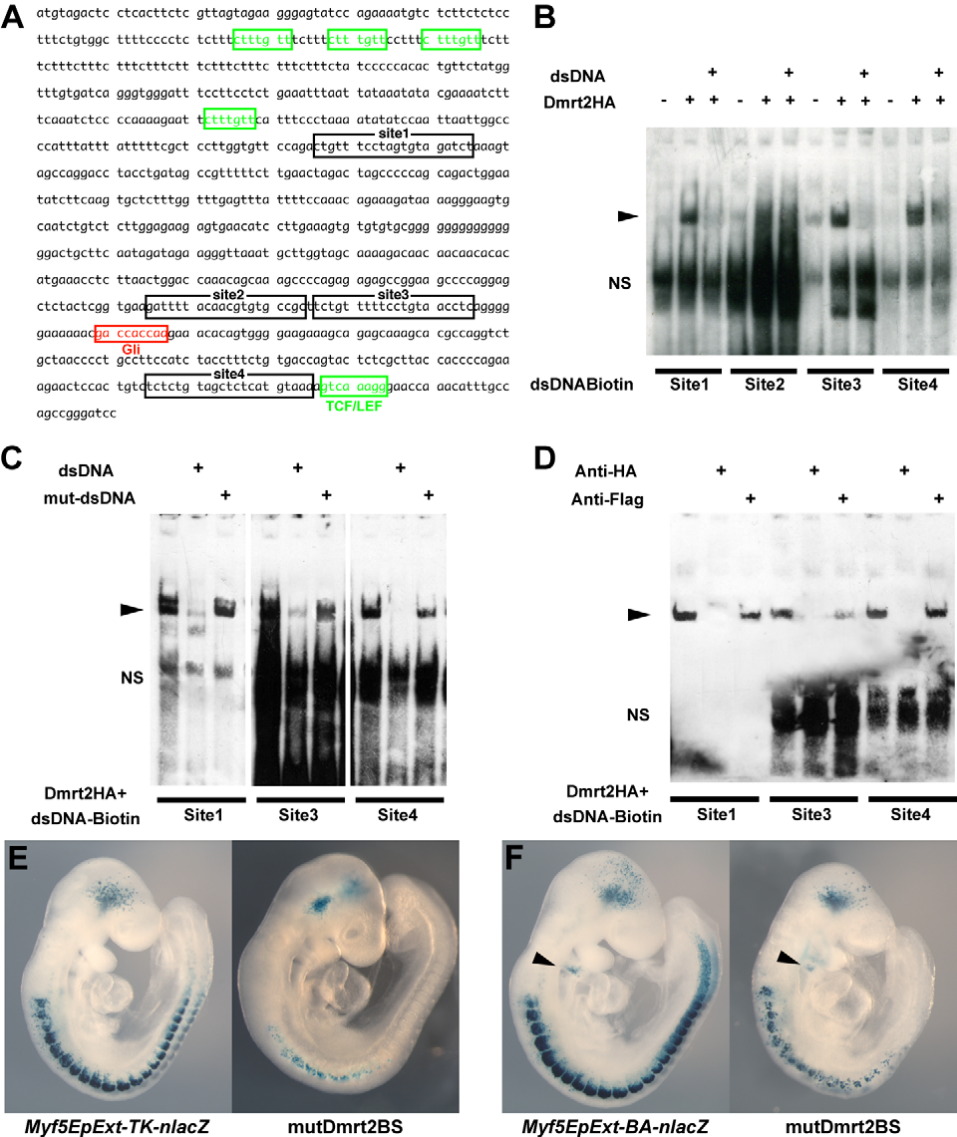
Dmrt2 binding sites in the *Myf5* epaxial enhancer are essential for normal activity *in vivo*. (A) The sequence of the *Myf5* epaxial enhancer (EpExt), which drives early *Myf5* expression in the epaxial domain of newly formed somites. TCF/LEF binding sites are boxed in green, the Gli1 binding site in red, and four putative Dmrt2 binding sites in black. (B–D) EMSA performed with oligonucleotides conjugated with biotin, containing Dmrt2 binding sites (1–4) as indicated in A, incubated with extracts of HEK293 cells, with (+) or without (−) a Dmrt2HA expression vector, or oligonucleotides without biotin (dsDNA) (including mutated oligonucleotides; mut-dsDNA). In the supershift assay (D), binding was disrupted by adding anti-HA (Monoclonal; Roche) to HA-tagged Dmrt2, whereas control anti-Flag antibody had no effect. The arrowheads show the binding between the oligos and the tagged protein. NS; non-specific. (E, F) Transient transgenic analysis to examine the role of the Dmrt2 binding sites in the *Myf5* EpExt enhancer. The transgenes, containing mutated Dmrt2 binding sites1, 3, 4, showed reduced β-galactosidase activity in developing somites with either the *TK* (E) or *Myf5-BA* (branchial arch) promoter region (F). The BA element that directs transgene expression to the branchial arches, provides a positive control (arrowheads in F). Observations on transgenic embryos are summarised in Table 1.

**Figure 6:**
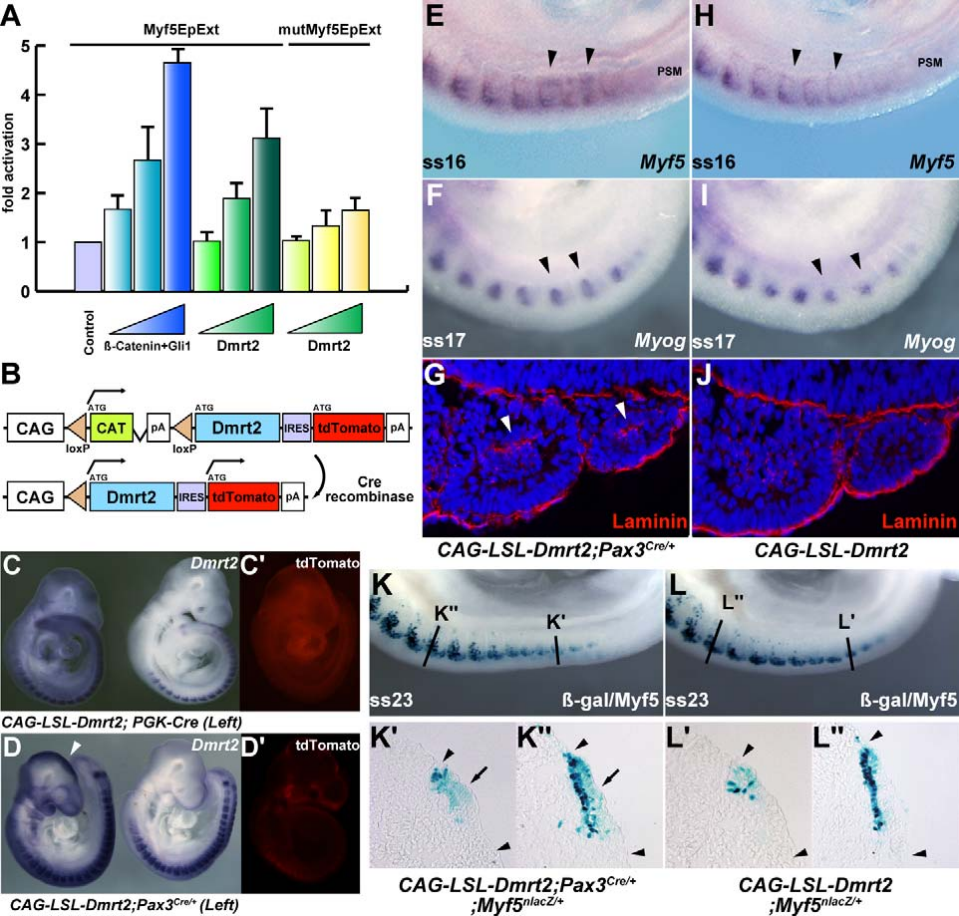
Overexpression of Dmrt2 accelerates myogenesis. (A) Luciferase assay in NIH3T3 cells with the *Myf5EpExt* fragment with (yellow bars) or without (green bars) mutated Dmrt2 binding sites 1, 3, 4 (mutMyf5EpExt) as used in Figure 5, and increasing concentrations of a Dmrt2-expression vector. As a positive control, β-catenin and Gli1 expression vectors were used (blue bars). Progressive increase in Dmrt2 expression, leads to increased activation of the *Myf5EpExt* which is lost when Dmrt2 binding sites 1, 3, 4 are mutated. (B) The transgene for *Dmrt2* conditional over-expression, in which Cre recombination removes the CAT sequence leading to Dmrt2 and reporter Tomato red expression under the strong CAG promoter. (C, C′, D, D′) Transgenic embryos, containing the *CAG-floxedstop-Dmrt2-IRES-tdTomato* (*CAG-LSL-Dmrt2*) transgene, crossed with *PGK-Cre* mice (left in C, C′), and *Pax3^Cre/+^* mice (left in D, D′). Ectopic *Dmrt2* expression is seen in *PGK-Cre* or *Pax3^Cre/+^* expressing embryos reflecting *Cre* expression (arrowheads in C, D). (E–I) Whole mount *in situ* hybridization of transgenic control or Pax3-Cre activated embryos at E9.5. *Myf5* (E.H) and *myogenin* (F, I) expression are up-regulated in developing somites of embryos overexpressing the *Dmrt2* transgene in Pax3 positive cells (arrowheads in E, F) compared to controls (H, I). (G, J) Sections showing somites of control or activated *Dmrt2* transgenic embryos at the same axial level, treated by immunohistochemistry with a laminin antibody. Laminin is detected in the premature myotome, where it is not normally present at this developmental stage. (arrowheads in G). (K, L) Whole mount X-gal staining of *Myf5^nlacZ/+^* embryos crossed onto the *Dmrt2* expressing transgenic line, with (K) or without (L) activation by Pax3-Cre, show perturbed location of β-galactosidase activity. This is shown on sections at the axial levels indicated in K and L, in which Pax3 activation of the *Dmrt2* transgene results in more extensive dermomyotome (arrows in K′, K″) and early myotome (K′) expression (not seen in the control (L′, L″). Arrowheads indicate somite extent.

The initiation of *Myf5* expression in the somite depends on an early epaxial enhancer [2],[3]. This element, used here in its extended form (*Myf5-EpExt*; [6]), is regulated by Gli and TCF binding sites, targets of Hedgehog and canonical Wnt signalling. The enhancer contains four sites that are close to the consensus for Dmrt2 binding ([28], Figure 5A). Of these, sites 1, 3 and 4 bind Dmrt2, as shown by electrophoretic mobility shift assays (Figure 5B–5D). This is specific since it is abolished by competition with the *Myf5* sequence, but not by this sequence with mutations in the Dmrt2 binding sites (Figure 5C). No good antibodies are available for Dmrt2 detection, so we used a human influenza hemagglutinin (HA) tagged version of the protein, and anti-HA antibodies to show that the complex is disrupted (Figure 5D). The *Myf5-EpExt* enhancer, with a *TK* or *Myf5BA* promoter region drives *nlacZ* transgene expression in somites. When the Dmrt2 sites are mutated in the *Myf5-EpExt* enhancer, this expression is very reduced in newly forming somites and is perturbed or absent in more mature somites (Figure 5E and 5F). Transactivation of the *Myf5-EpExt* enhancer by Dmrt2 was shown by co-transfection experiments in NIH3T3 cells, with this enhancer driving a luciferase reporter and increasing amounts of a Dmrt2 expression vector; mutation of the Dmrt2 sites (1,3,4) in the *Myf5-EpExt* sequence showed that activity depends on Dmrt2 binding (Figure 6A). Strong transactivation by Dmrt2 was also seen in HEK293 cells (results not shown). In order to look at Dmrt2 activation *in vivo*, a transgene was constructed in which expression of a sequence encoding Dmrt2, under the transcriptional control of a strong universal *CAG* promoter, depends on Cre recombinase removal of an intervening *CAT* sequence. When crossed with a *PGK-Cre* line [32], *Dmrt2* expression is seen throughout the embryo (Figure 6C, the left hand embryo), as also evidenced by expression of the Tomato red reporter (Figure 6C′). When crossed with a *Pax3^Cre/+^* line [33], the *Dmrt2* transgene is transcribed at sites of Pax3 expression, including the somites and dorsal neural tube (Figure 6D), where Tomato red coloration is also detected (Figure 6D′). Expression of the endogenous *Myf5* gene was monitored in these transgenic embryos at E9.5 (ss 23). When the *Dmrt2* transgene is activated by Pax3-Cre, *Myf5* expression is more extensive in the newly formed somites (Figure 6E) than in the control embryo (Figure 6H). *Myogenin* transcripts are also more widely expressed (Figure 6F and 6I) and sections of an immature somite show premature presence of assembled laminin, which marks the myotome basement membrane (Figure 6G, compared to Figure 6J). Together, these data indicate that myogenesis is accelerated in these embryos, leading to an earlier entry and organisation of cells in the myotomal space. The effect on *Myf5* expression is also seen when the *Dmrt2* transgene is expressed in *Myf5^nlacZ/+^* mice (Figure 6K, compared to Figure 6L) and sections show that *Myf5* is now ectopically expressed in the central/hypaxial dermomyotome in the presence of Pax3-Cre (Figure 6K′ and 6K″ compared to Figure 6L′ and 6L″). A similar result was seen with PGK-Cre (Figure S3). This therefore demonstrates that Dmrt2 activates the *Myf5* gene *in vivo* in the somite, leading to ectopic expression. Acceleration of the onset of myogenesis and myotome formation are probably the consequence of over-expression of Dmrt2 and over-activation of the epaxial enhancer of *Myf5*.

## Discussion

**Figure 7:**
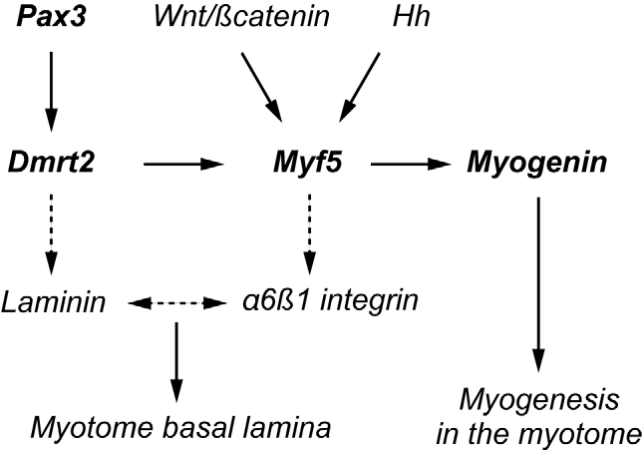
A schematic representation of the regulatory network presented in this paper. Direct targets are shown in heavy type. Indirect targets are indicated by a discontinuous arrow. The *myogenin* promoter depends on a critical Ebox [47],[48] targeted by Myf5. α6β1 integrin expression depends on Myf5, in cells entering the myotome [39]. This receptor interacts with Laminin1 as a ligand, leading to formation of the basement membrane of the myotome. Laminin expression is modulated by Dmrt2 (see also [25]).

We establish a genetic network that is initiated in the dermomyotome by Pax3 regulation of *Dmrt2* and which, through Dmrt2 regulation of *Myf5*, orchestrates the onset of myogenesis in the myotome. This direct transcriptional cascade and further genetic targets discussed in this paper are presented schematically in Figure 7.

Our demonstration that *Dmrt2* is a Pax3 target was initially based on a genetic analysis that showed up-regulation of this gene in the presence of PAX3-FKHR. This transcriptional activator of Pax3 target genes may introduce a bias due to the FKHR domain, however down-regulation of *Dmrt2* in the presence of the Pax3-Engrailed fusion protein and in the absence of Pax3 reinforces the interpretation. This is confirmed by the identification of a Pax3 dependent regulatory sequence, located at 18 kb 5′ of the *Dmrt2* gene, which directs transgene expression to the somite. Mutation of the Pax3 site shown to bind this factor *in vitro* and *in vivo*, abolishes most of the activity in the dermomyotome. Since Pax7 can replace Pax3 in the trunk [34], it is probable that this site also binds Pax7. Indeed in *Pax3* mutants, *Dmrt2* continues to be expressed in the remaining dermomyotome [26], where Pax7 prevents cell death in the absence of Pax3 [1]. Expression of the transgene is not confined to the dermomyotome, suggesting that the 286 bp element also responds to factors in the ventral somite, where transcription of the endogenous gene is repressed by other regulatory sequences. *Dmrt2* is present in a locus that includes *Dmrt1* and *Dmrt3*. The latter lies about 30 kb 5′ of the 286 bp sequence. However, *Dmrt3* is not transcribed in the somites and there is no indication that this regulatory element directs any aspect of *Dmrt3* expression, which is characteristically seen at sites in the head [35],[36] for example, where no labelling with the *Dmrt2-286-TKnlacZ* transgene is observed. Other regulatory regions, namely 2.6 kb immediately 5′ of *Dmrt1* which directs transgene expression to the testis [37] or a highly conserved sequence between *Dmrt1* and *Dmrt3*, associated with XY sex reversal in humans [38], which directs *Dmrt3*-like transgene expression, also show regulatory properties characteristic of the adjacent downstream gene only.

It is well established that the DM domains of Dmrt2 factors bind to a similar consensus sequence [28], however direct target genes have not been identified. We now show that the *Myf5* gene is targeted by Dmrt2 through the expected DM consensus sequence. The transcriptional activity of mammalian Dmrt factors is not well defined, in contrast to *Drosophila* Dsx which acts as a transcriptional activator or repressor, when produced from a male or female allele, respectively [29]. We demonstrate that Dmrt2 functions as a transcriptional activator on a DM consensus binding site in the *Myf5* early epaxial element. This was observed in assays with the NIH3T3 fibroblast cell line, and with HEK293 cells in which transcriptional activity of Dmrt1 was not detectable under conditions in which a Dmrt1-VP16 fusion protein transactivated a luciferase reporter through DM sites [28]. Our transgenic experiments which lead to Dmrt2 over-expression *in vivo* also point to the action of this factor as an activator of *Myf5* expression.

*Dmrt2* mutant phenotypes in which somite disorganisation and effects on myogenesis had been documented previously, focussed on E10.5 and E11.5 embryos [25],[26]. We observed that *Dmrt2* transcription begins to be down-regulated from E10.5 and transcripts are no longer detectable in most somites by E11.5. The muscle recovery noted in *Dmrt2* mutants at later stages [25] is probably due to the invasion of the underlying muscle by Pax3/7 positive cells, from the central dermomyotome, which takes place from E10.5 [7]. Indeed in *Dmrt2* mutants at E10.5, the absence of apically polarised N-cadherin, in the dermomyotome ([25], Figure 4), may indicate de-epithelialization and premature disaggregation of the central domain which is the source of these muscle progenitor cells. We have concentrated our analysis on the onset of myogenesis when *Dmrt2* is strongly expressed in the dermomyotome. In the absence of Dmrt2, the onset of *Myf5* expression is affected. This depends on the epaxial enhancer [2],[3], which we show is regulated through Dmrt2 binding sites. This enhancer is activated in the epaxial somite by signals from the adjacent axial structures [6]. The requirement for Dmrt2 will restrict this activation to the dermomyotome. Dmrt2 alone does not direct ectopic expression of *Myf5* outside the somite, indicating that other factors also play a role in this restriction. Myf5 plays a key role in the initiation of myogenesis, manifested by the formation of the early epaxial myotome. The perduration of β-galactosidase, either from a *Myf5^nlacZ^* allele or from a *Myf5-EpExt* regulated transgene, marks these first myogenic cells. When *Myf5* expression is perturbed by the lack of Dmrt2, the formation of the myotome is affected. In contrast, over-expression of Dmrt2 promotes *Myf5* expression and myotome formation. Activation of the myogenic differentiation programme is evidenced by expression of the myogenic differentiation gene, *myogenin*, which is delayed in somites in the *Dmrt2* mutant and increased in the early myotome when Dmrt2 is over-expressed. An important aspect of myotome formation is the laying down of the basal lamina that confines this somite compartment. In the absence of Dmrt2, the expression of myogenic markers, such as myogenin, in the myotome is more diffuse ventrally and this correlates with a lack of laminin [25]. In contrast, when *Dmrt2* is over-expressed, we observed premature expression of laminin, associated with an acceleration of myotome formation. Laminin is the ligand of α6β1 integrin, required for the formation of the myotome basal lamina. Interestingly, α6β1 integrin is not expressed on myogenic cells delaminating from the dermomyotome in *Myf5* mutant mice, in which cells fail to locate correctly and the early myotome does not form [39]. Since this early expression of *Myf5* depends on the epaxial enhancer, *Dmrt2* lies genetically upstream of both this integrin receptor and its laminin ligand (Figure 7).

The *Pax3-Dmrt2-Myf5* regulatory cascade, identified by genetic and molecular approaches, has consequences for the onset of skeletal muscle formation, both in terms of the activation of the myogenic regulatory programme and of the organisation of myogenic cells and their progenitors within the somite. In *Pax3* mutants, epaxial myogenesis occurs, however perturbations in epaxial derivatives, such as deep back muscles, are observed [40]. Other *Myf5* regulatory sequences control myotomal expression [2]. Pax3 also directly activates another *Myf5* regulatory element at later developmental stages [9]. This key factor therefore, directly or indirectly influences different spatiotemporal aspects of myogenesis. Expression of *Dmrt2* is not limited to myogenic progenitors in the epaxial domain, but is present throughout the developing dermomyotome, where it may be implicated in maintaining this epithelial structure [25]. We have recently identified *Foxc2* as a gene that is negatively regulated by *Pax3* in the dermomyotome and that promotes non-myogenic cell fates [41]. Identification of these targets begins to reveal how Pax3 controls cell behaviour in the somite before the determination of myogenic cells. Integration of Pax3 targets into a regulatory network, comprising elements such as those discussed in this paper (Figure 7), is essential for understanding how such a key regulatory factor orchestrates the progression from stem cell towards differentiated tissue. Defining such regulatory networks that control developmental processes is a major challenge, taken up by the emerging field of systems biology.

## Materials and Methods

### Mice

Generation and genotying of *Pax3^IRES-nlacZ/+^*, *Pax3^GFP/+^* [7], *Pax3^PAX3FKHR-IRESnlacZ/+^* [31], *Pax3^Pax3-En-IRESnlacZ/+^* [9], *Pax3^Cre/+^* [33], *PGK-Cre* [32], *Myf5^nlacZ/+^* [42], *Dmrt2^+/−^*, and *Dmrt2^−/−^* [25] mice was carried out as previously described.

### Screening

*Pax3^GFP/+^* mice were crossed with *PGK-Cre* transgenic mice to obtain *Pax3^GFP/+^; PGK-Cre* females, and these females were crossed with *Pax3^PAX3FKHR-IRESnlacZ/+^* males to obtain embryos with one *Pax3^GFP^* allele and one floxed *Pax3^PAX3FKHR-IRESnlacZ^* allele [31]. Somites were dissected from the interlimb region of E9.5 embryos. The GFP positive cells were collected by FACs from *Pax3^GFP/+^* and *Pax3^Pax3FKHR–IRESnlacZ/GFP^* mutant embryos. About 3.0×10^5^ cells were collected for RNA preparation, cDNA synthesis and subsequent analysis using Affymetrix microarrays. Detailed description of the microarray analysis will be published elsewhere (Lagha and Sato, in preparation).

### Plasmid construction and generation of transgenic mice

The mouse *Dmrt2* cDNA (contains the complete coding region; NM145831) was isolated by RT-PCR from cDNA of RNA prepared from C57BL/6 mouse embryos at E9.5. The *Dmrt2* cDNAs with or without a 3′ sequence coding HA tag were subcloned into pBS and pcDNA3 vectors for *in situ* hybridization, electrophoresis mobility shift, and overexpression assays.

To generate transgenes with the conserved 286 bp sequence located at −18 kb from the *Dmrt2* gene (Dmrt2-286), or the extended *Myf5* epaxial enhancer (*Myf5-EpExt*, [6]), these sequences were obtained by PCR from total DNA of E9.5, C57BL/6 embryos using KOD DNA polymerases (Novagen) and ligated into pBS plasmids to lie 5′ of *TK-nlacZ* or *Myf5BA-nlacZ* transgenic sequences [43]. The primers used were as follows, Dmrt2-286-Fwd; 5′-GCAGCGGCCGCTATCAGGAATGAATTAGCTTGTTTCCCTCC-3′, Dmrt2-286-Rev; 5′- GCCACTAGTCTGGACCTCCTTTCCCCGATGCGTGCCCT-3′, Myf5-EpExt-Fwd; 5′-GCAGCGGCCGCATGTAGACTCCTCACTTCTCGTTAGTAGA-3′, Myf5-EpExt-Rev; 5′- GCCACTAGTGGATCCCGGCTGGCAAATGTTTGGTTCCCT-3′.

Transgenes with mutated Pax3 or Dmrt2 binding sites were created with the QuikChange Multi Site-Directed Mutagenesis kit (Stratagene). The *Myf5EpExt-TK* fragment was digested with NheI and NcoI, and ligated into the pGL4 vector (Promega) in front of the luciferase reporter for transactivation assays.

For transgenic expression of Dmrt2, the HindIII and BamHI fragment digested from the *pBS-CAG-floxedCAT* vector [44], the HindIII and XbaI fragment digested from *pBS-Dmrt2*, and the XbaI and BamHI fragment digested from the *pCMVTnT-IRES2-tdTomato-pA* vector (*pCMVTnT*; Promega, *pIRES2*; Clontech, *tdTomato*; a gift from Dr. Tsien) were all ligated together to make the *pBS-CAG-floxedCAT-Dmrt2-IRES2-tdTomato* vector (*pBS-CAG-LSL-Dmrt2*). *pBS-Dmrt2-286-TK-nlacZ*, *pBS-Myf5EpExt-TK-nlacZ*, *pBS-Myf5EpExt-BA-nlacZ* and *pBS-CAG-LSL-Dmrt2* vectors were linearized to produce transgenic mice according to standard techniques. The primers used for genotyping of *CAG-LSL-Dmrt2* mice were as follows, F502; CTCCGGAGGCAGCAGGCCACAG, R620; ATGCTTTTGGCCAGCAAACTCG; R794; CGCGATGTCCCAAATGGACCTAA (wildtype; 293 bp, transgene; 119 bp).

For BAC transgenic analysis, a BAC containing *Dmrt2* genomic DNA (−150 kb/+50 kb: clone RP24-290E12 purchased from BACPAC resources center, CHORI) was used for targeting with an *nlacZ* reporter into the *ATG* site of *Dmrt2*. The *Dmrt2* BAC with a mutated Pax3 binding site in the 286 bp sequence was created using the fragment from the transgene with the mutated Pax3 binding site in *Dmrt2-286*. All BAC recombineering were performed with SW105 and SW106 strains [45].

### Whole mount staining of embryos and immunohistochemistry

Whole mount *in situ* hybridization analyses and X-gal staining were performed as previously described [15]. For double detection of β-galactosidase (β-gal) from a *Pax3 nlacZ* allele and *Dmrt2* transcripts, the former was visualized by Red-gal (Sigma), and the latter using a digoxigenin (DIG)-labeled probe visualized with BM purple (Roche). For immunohistochemistry of laminin, the antibody against laminin (Chemicon; AL-4 clone) was used as described [39].

### Electrophoretic mobility shift assays (EMSA)

EMSA was performed using the LightShift Chemiluminescent EMSA kit (Thermo Fisher Scientific). Protein extracts containing Pax3 or Dmrt2 protein were prepared from HEK293 cells transfected with *pcDNA3-Pax3* [46] or *pcDNA3-Dmrt2HA*. Cells were then lysed on ice in extraction buffer (15 mM Tris pH 7.4; containing protease inhibitor cocktail (Roche)) supplemented with 0.5% NP40. Lysates were centrifuged at 2000×g for 5 min at 4°C and the supernatant was stored in 25% glycerol at −80°C until ready for use. The 5′-biotin-conjugated oligos and unlabeled probes used were as follows, Pax3BS1; 5′-CTTGTTTCCCTCCTGTAAGTGATCC-3′, Pax3BS2; 5′-CTGTGGTGTGTGACTAATGGAGTGC-3′, Pax3BS3; 5′-CTCTCAGGGCTGGTTTAAGACTCA-3′, Pax3BS4; 5′-CATAGCAGGGACACAGTAAAGCCAC-3′, mutPax3BS2; 5′- CTGTGGTGACGTCTAAATGGAGTGC-3′, Dmrt2BS1; 5′-CTGTTTCCTAGTGTAGATCT-3′, Dmrt2BS2; 5′- GATTTTACAACGTGTGCCGC-3′, Dmrt2BS3; 5′-TCTGTTTTTCCTGTAACCTC-3′, Dmrt2BS4; 5′-TCTCTGTAGCTCTCATGTAAA-3′, mutDmrt2BS1; 5′-CTGTTTCCTTGTTTTGATCT-3′, mutDmrt2BS3; 5′-TCTGTTTTTCCTTTTATCTC-3′, mutDmrt2BS4; 5′-TCTCTTTTGCTCTCATTTTAA-3′. Sense and anti-sense strands were synthesized and 40 fmol biotin-labeled double-stranded DNA was mixed with 1 µg protein from a HEK293 cell extract. Gel mobility shift assays and hybridization were performed according to the instructions of Invitrogen, using 6% DNA retardation gels (Invitrogen).

### Chromatin Immunoprecipitation (ChIP)

For ChIP experiments, somites were collected from E9.5 embryos with heads, neural tubes and internal organs removed. These samples were mechanically digested through a syringe and dissociated cells were fixed with 1% formaldehyde at room temperature for 10 min. The ChIP procedure was performed according to an enzymatic digestion protocol (ChIP-IT Express; ActiveMotif) with Pax3 antibody raised from Rabbit (ActiveMotif and Geneka; Bajard et al., 2006 and Lagha et al., 2008) or normal Rabbit serum (Sigma) as a control. Input DNA and immunoprecipitated DNA were analyzed by PCR. The sequences of primers were designed as follows. The *Dmrt2*-18 kb conserved region (286 bp) Fwd; 5′-GCTTGTTTCCCTCCTGTAAGT-3′, Rev; 5′- GTGTAGGATCTGTGGCTTTAC-3′ and the *Dmrt2*+20 kb control conserved region (+20 kb) Fwd; 5′-GGTTCTCATAATTTACATGCT-3′, Rev; 5′- TCCAACATCTGATTGTACTTA-3′.

### Luciferase assay

For luciferase assays, *pGL4-Myf5EpExt* vectors were transfected into NIH3T3 cells together with a *pRL-TK* plasmid (Promega; for normalizing) and test plasmids (Gli1 and β-catenin expression vectors were kindly provided by Dr. S. Brunelli). Transfected cells were cultured for 24 hours, and subjected to luciferase assays using the Dual-Luciferase Reporter Assay System (Promega).

**Table 1:** Transient transgenic embryos and lines (where indicated) (at E9.5, unless otherwise indicated).

Transgene	Number of embryos	*nlacZ* expression
*Dmrt2-286-TK-nlacZ*	5	in dermomyotome and ventral somites (3) – as in Figure 3E in dermomyotome and myotome (2)
*Dmrt2-286 (mutPax3BS)-TK-nlacZ*	4	major loss in dermomyotome expression (3) – as in Figure 3F ectopic expression, also detected in head (1)
*Dmrt2 BAC-nlacZ*	2 (lines)	In dermomyotome and myotome (2)
*Dmrt2 BAC (mutPax3BS)-nlacZ*	2 (lines)	Major loss of somitic expression (2)
	4	Major reduction in somite expression, with labelling mainly detectable in caudal dermomyotome (3 at E8.5, 1 at E9.5)
*Myf5EpExt-TK-nlacZ*	3	early epaxial somites (3) – as Figure 5E left panel
*Myf5EpExt(mutDmrt2BS)-TK-nlacZ*	6	weak in epaxial somites (4) – as Figure 5E right panel only in head (2)
*Myf5EpExt-BA-nlacZ*	2	BA and epaxial somites (2) – as Figure 5F left panel
*Myf5EpExt(mutDmrt2BS)-BA-nlacZ*	4	Weak in epaxial somites (2) – as Figure 5F right panel only in BA (2)

The numbers in brackets indicate the total number of transgenic embryos examined. BA; branchial arch.

## Supporting Information

Figure S1 The expression of *Dmrt2* is controlled by Pax3. Whole mount *in situ* hybridization with a *Dmrt2* probe of *Pax3^GFP/+^* (left) and *Pax3^GFP/nLacZ^* (right) embryos at E9.25. The number of somites (ss, somite stage) is indicated on each panel. When Pax3 is absent, *Dmrt2* transcripts are reduced, notably in the hypaxial and epaxial domains where *Pax7* is not expressed.
(0.58 MB TIF)

Figure S2 *Dmrt2* BAC transgenics show that the conserved Pax3 binding site is important for regulation of *Dmrt2* expression in the somite. A BAC containing Dmrt2 genomic DNA (−150 kb/+50 kb), including the conserved 286 bp *Dmrt2* element, (clone RP24-290E12 purchased from BACPAC resources center, CHORI) was targeted with an *nlacZ* reporter. (A) A transgenic embryo (E8.5) with this BAC shows dermomyotome expression. (B) When the Pax3 binding site is mutated in the context of this BAC, transgene expression in the somite is severely reduced. In (A), a transgenic line is shown. In (B), a transitory transgenic (F0) embryo is shown, where residual expression is observed. Two lines showed no expression (see Table 1).
(1.47 MB TIF)

Figure S3 Ubiquitous overexpression of Dmrt2 shows *Myf5* upregulation only in somites. Transgenic embryos, containing the *CAG-floxedstop-Dmrt2-IRES-tdTomato* (*CAG-LSL-Dmrt2*) transgene, crossed with *PGK-Cre* mice (left). Whole mount *in situ* hybridization of transgenic control or PGK-Cre activated embryos at E9.5. *Myf5* expression is up-regulated in developing somites of embryos where *Dmrt2* is overexpressed (arrowhead in left panel) compared to controls (right).
(0.82 MB TIF)
