# Supplementary figures and images for: A Pax3/Dmrt2/Myf5 Regulatory Cascade Functions at the Onset of Myogenesis
Source: PLoS Genet. 2010 Apr 1;6(4):e1000897. doi: 10.1371/journal.pgen.1000897 (PMC2848553; doi:10.1371/journal.pgen.1000897)

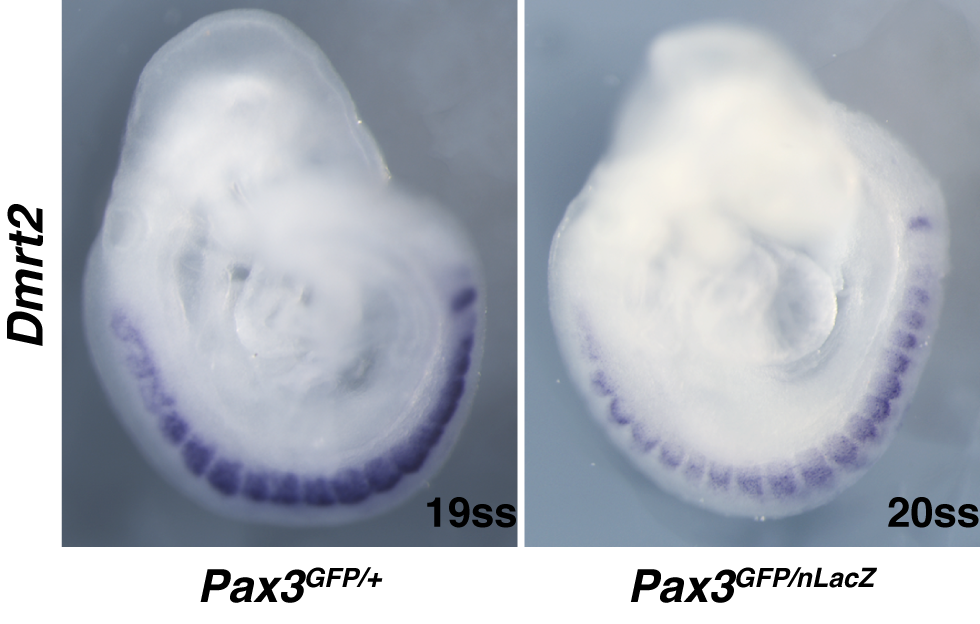

Supplement: Figure S1 — The expression of Dmrt2 is controlled by Pax3. Whole mount in situ hybridization with a Dmrt2 probe of Pax3GFP/+ (left) and Pax3GFP/nLacZ (right) embryos at E9.25. The number of somites (ss, somite stage) is indicated on each panel. When Pax3 is absent, Dmrt2 transcripts are reduced, notably in the hypaxial and epaxial domains where Pax7 is not expressed. (0.58 MB TIF) [file pgen.1000897.s001.tif]

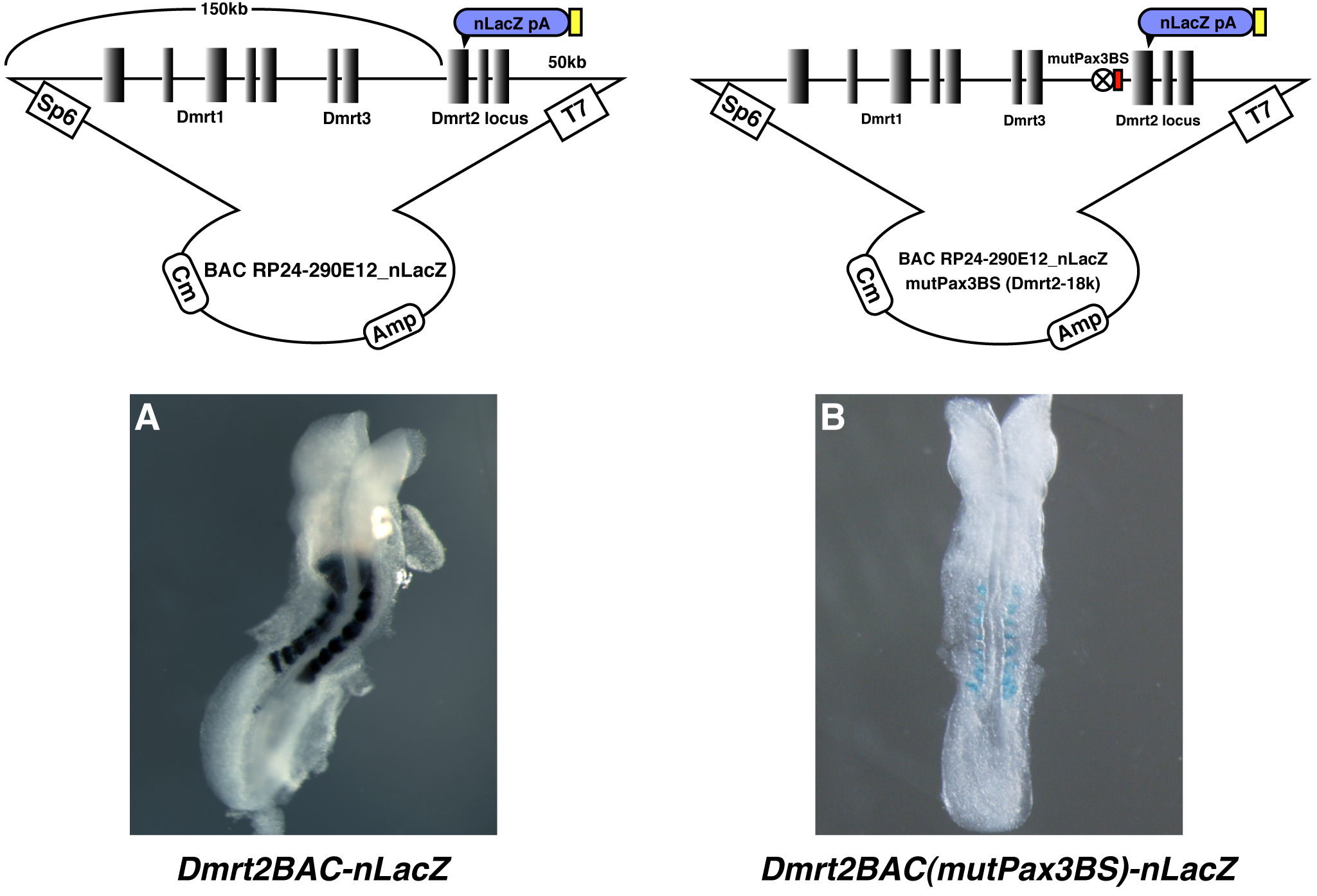

Supplement: Figure S2 — Dmrt2 BAC transgenics show that the conserved Pax3 binding site is important for regulation of Dmrt2 expression in the somite. A BAC containing Dmrt2 genomic DNA (−150 kb/+50 kb), including the conserved 286 bp Dmrt2 element, (clone RP24-290E12 purchased from BACPAC resources center, CHORI) was targeted with an nlacZ reporter. (A) A transgenic embryo (E8.5) with this BAC shows dermomyotome expression. (B) When the Pax3 binding site is mutated in the context of this BAC, transgene expression in the somite is severely reduced. In (A), a transgenic line is shown. In (B), a transitory transgenic (F0) embryo is shown, where residual expression is observed. Two lines showed no expression (see Table 1). (1.47 MB TIF) [file pgen.1000897.s002.tif]

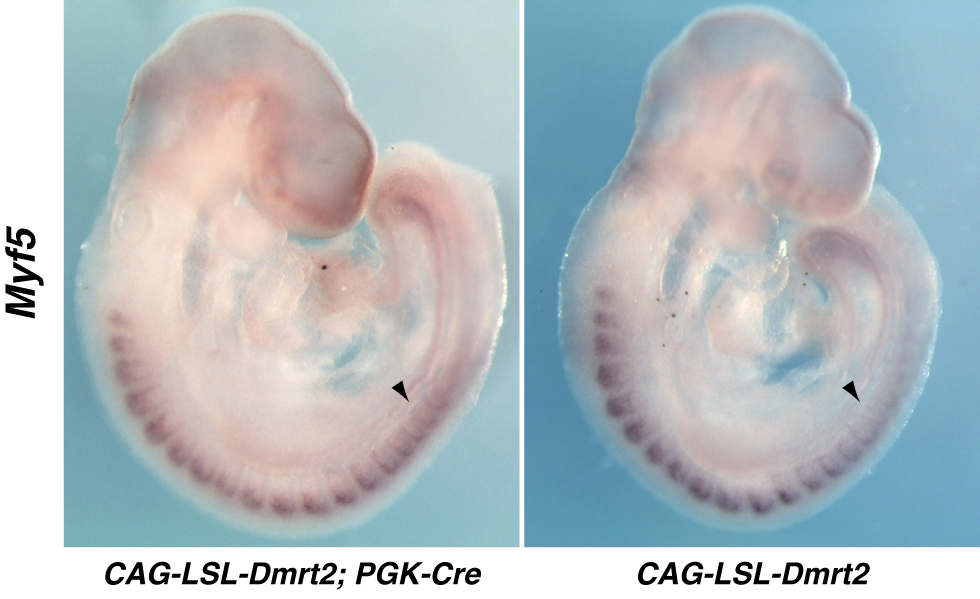

Supplement: Figure S3 — Ubiquitous overexpression of Dmrt2 shows Myf5 upregulation only in somites. Transgenic embryos, containing the CAG-floxedstop-Dmrt2-IRES-tdTomato (CAG-LSL-Dmrt2) transgene, crossed with PGK-Cre mice (left). Whole mount in situ hybridization of transgenic control or PGK-Cre activated embryos at E9.5. Myf5 expression is up-regulated in developing somites of embryos where Dmrt2 is overexpressed (arrowhead in left panel) compared to controls (right). (0.82 MB TIF) [file pgen.1000897.s003.tif]
